# Transcriptional co-expression regulatory network analysis for Snail and Slug identifies *IL1R1*, an inflammatory cytokine receptor, to be preferentially expressed in ST-EPN-*RELA* and PF-EPN-A molecular subgroups of intracranial ependymomas

**DOI:** 10.18632/oncotarget.26211

**Published:** 2018-10-26

**Authors:** Prit Benny Malgulwar, Vikas Sharma, Ashutosh Singh Tomar, Chaitenya Verma, Aruna Nambirajan, Manmohan Singh, Vaishali Suri, Chitra Sarkar, Mehar Chand Sharma

**Affiliations:** ^1^ Department of Pathology, All India Institute of Medical Sciences, New Delhi-110029, India; ^2^ Center for Cellular and Molecular Biology–Council of Scientific and Industrial Research (CCMB-CSIR), Hyderabad, Telangana-500007, India; ^3^ Department of Neurosurgery, All India Institute of Medical Sciences, New Delhi-110029, India

**Keywords:** ependymoma, ST-EPN-RELA and PF-EPN-A molecular groups, co-expression study, Snail and Slug, *IL1R1*

## Abstract

Recent molecular subgrouping of ependymomas (EPN) by DNA methylation profiling has identified ST-EPN-*RELA* and PF-EPN-A subgroups to be associated with poor outcome. Snail/Slug are cardinal epithelial-to-mesenchymal transcription factors (EMT-TFs) and are overexpressed in several CNS tumors, including EPNs. A systematic analysis of gene-sets/modules co-expressed with *Snail* and *Slug* genes using published expression microarray dataset (GSE27279)identified 634 genes for *Snail* with enriched TGF-β, PPAR and PI3K signaling pathways, and 757 genes for *Slug* with enriched focal adhesion, ECM-receptor interaction and regulation of actin cytoskeleton related pathways. Of 37 genes commonly expressed with both *Snail* and *Slug*, *IL1R1*, a cytokine receptor of interleukin-1 receptor family, was positively correlated with Snail (r=0.43) and Slug (r=0.51), preferentially expressed in ST-EPN-*RELA* and PF-EPN-A molecular groups, and enriched for pathways related to inflammation, angiogenesis and glycolysis. *IL1R1* expression was fairly specific to EPNs among various CNS tumors analyzed. It also showed significant positive correlation with EMT, stemness and MDSC (myeloid derived suppressor cell) markers. Our study reports *IL1R1* as a poor prognostic marker associated with EMT-like phenotype and stemness in EPNs. Our findings emphasize the need to further examine and validate IL1R1 as a novel therapeutic target in aggressive subsets of intracranial EPNs.

## INTRODUCTION

Ependymomas (EPN) are uncommon gliomas that recapitulate the ependymal cells lining the ventricles and spinal cord [1]. They occur throughout the neuraxis and are the third most common central nervous system (CNS) neoplasm in children [1]. Surgical resection is the treatment of choice with limited role for adjuvant radiotherapy [2, 3]. However, despite advances in neurosurgical techniques and higher rates of gross total excision, there has been little change in the overall and progression free survival of intracranial EPNs [4]. Histologically similar EPNs show site-specific differences in biological behavior and are postulated to arise from site-specific subsets of radial glia-like stem cells [5]. Large scale DNA methylation profiling has delineated nine molecular subgroups that predict outcome better than World Health Organisation (WHO) histological grade [1, 6]. Among these, the two largest subgroups: ST-EPN-*RELA* and PF-EPN-A, represented by supratentorial (ST) WHO Grade II/III EPNs harboring *RELA* fusions and posterior fossa (PF) WHO Grade II/III childhood EPNs with a CpG island methylator phenotype (CIMP) respectively, are associated with extremely poor outcomes [1, 6–8]. Further insights into the pathogenesis of these enigmatic tumors, particularly of the aggressive subgroups, are necessary to identify newer prognostic markers and therapeutic targets and thereby, devise alternate treatment modalities.

Epithelial-to-Mesenchymal Transition (EMT) is an evolutionary conserved physiological and developmental process that results in a series of rapid changes in cellular phenotype [9]. During EMT, epithelial cells down-regulate cell-cell adhesion, alter polarity and reorganize cytoskeletal structures to become disengaged from surrounding cells and acquire motility and invasiveness [9, 10]. In cancer, the process of EMT confers enhanced metastatic potential, increases stem cell-like characteristics, inhibits apoptosis and aids in immune escape, all of which are fundamental events in tumor progression [9–13]. The cardinal EMT regulating transcription factors (TFs): Snail and Slug have been found to be upregulated in various cancers [14–22], including ependymomas [23]. However, the mechanisms by which Snail and Slug contribute to the pathogenesis and aggressiveness of these tumors are largely unexplored.

Co-expression analysis of genes has recently emerged as a powerful tool for multi-gene analysis of large scale datasets [24]. While comparisons of gene expression datasets list out differentially expressed genes, distinguishing the functionally important genes remains challenging. Clarke C et al described the concept of gene co-expression analysis as ‘guilt-by-association’, wherein the groups of genes (also known as co-expressed modules) that maintain a consistent co-expression pattern likely share a common biological role and functional importance [25]. Co-expression analysis tools have been widely used in numerous biological investigations [26], including cancer biology. EGFR and PDGFR gene co-expression modules identified molecular subgroups of gliomas with distinct genomic/transcriptomic patterns and clinical outcome [27]. A TCGA-Glioma based co-expression study for CD133 and CD44 in glioblastomas (GBM) reported that CD133 module tumors were enriched for the Proneural GBM subtype, while CD44 module tumors were enriched in Mesenchymal subtype [28]. Another TCGA expression based study in Grade II and III oligodendrogliomas identified a co-expression network of six mitosis-regulating genes: *AURKA, NDC80, CENPK, KIAA0101, TIMELESS* and *MELK* that was found to have association with histological grade, TCGA subtype, proliferative indices and patient outcome [29]. Similar studies are lacking in ependymomas and we aimed to perform gene co-expression analysis for Snail and Slug genes in EPNs to explore and identify potential genes of pathogenic significance.

## RESULTS

A total of 75 in-house samples of Grade II/III ependymomas were included in the study, subdivided into five clinico-pathologic-molecular subgroups, viz. ST-RELA+, ST-RELA-, PF-A, PF-B and SP, based on site, *C11orf95-RELA* fusions (in ST EPNs), and age (in PF EPNs). The clinicopathological features are summarized in Supplementary Table 1. *YAP1* fusions were not detected in any of the ST EPNs tested.

### Gene expression analysis identified consistent upregulation of *Snail* and *Slug* in intracranial EPNs across independent cohorts

**Figure 1:**
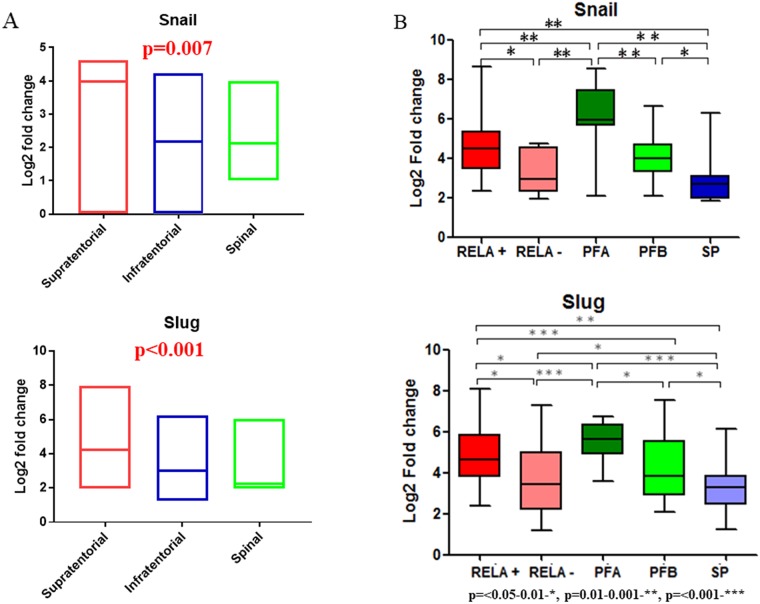
Gene expression analysis for Snail and Slug using published ependymoma cohorts and qRT-PCR data (in-house) in relation to molecular subgroups Box plots for gene expression analysis of Snail and Slug in supratentorial, infratentorial and spinal compartments **(A)**. Realtime Quantitative PCR analysis for Snail and Slug expression in different molecular subgroups of in-house ependymomas **(B)**.

Analysis of the three gene expression microarray datasets of EPNs: GSE27279 [7], GSE21687 [30] and GSE50385 [31], revealed overexpression of *Snail* and *Slug* in intracranial as compared to spinal EPNs (p=0.002) (Figure 1A). qRT-PCR for *Snail* and *Slug* on the 75 in-house EPNs showed higher expression levels of Snail and Slug in ST-RELA+ subset as compared to ST-RELA-, and higher expression levels in pediatric PF EPNs (PF-A) as compared to PF-B group, while SP group showed minimal upregulation of these transcription factors (Figure 1B).

### Gene co-expression network analysis and its functional annotation identifies distinct cellular functions regulated by *Snail* and *Slug*

**Figure 2:**
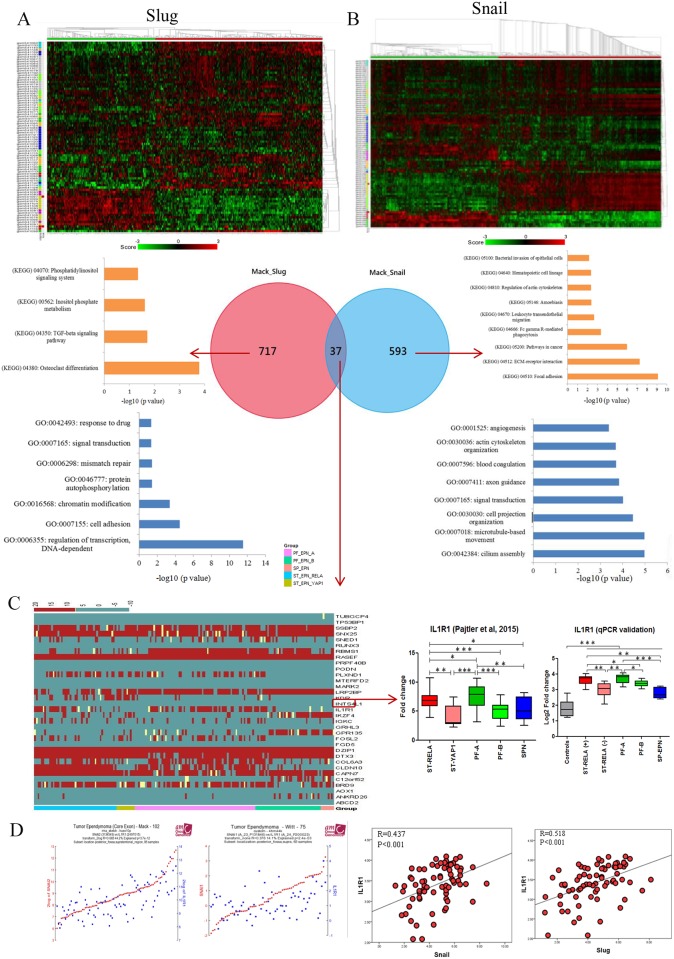
Co-expression network analysis of Snail and Slug and identification of IL1R1 Heatmap and Venn diagram showing different co-expressed genes with its pathway analysis for Snail and Slug **(A and B)**, Heatmap for 37 co-expressed genes between Snail and Slug and expression of IL1R1 in published dataset (Pajtler KW, et al. 2015) and in-house cohort **(C)**, Correlation plots of IL1R1 expression in published dataset and in-house cohort with Snail and Slug expression **(D)**.

Gene co-expression network analysis using R2 software on GSE27279 dataset [7] generated a total of 634 and 757 genes for *Snail* (positive=241, negative=393) and *Slug* (positive=364, negative=393) respectively (Figure 2A and 2B), of which only 37 genes were common to both gene-sets, indicating limited number of genes that are co-regulated by both *Snail* and *Slug* (Figure 2B). Among 593 genes co-regulated only by *Snail*, KEGG (Kyoto Encyclopedia of Genes and Genomes) pathway analyzing tool identified Osteoclast differentiation (p= 0.0001), TGF-β signaling pathway (p= 0.019) and Inositol phosphate metabolism (p= 0.024) as the enriched pathways (Figure 2B, Supplementary Table 2A). In biological processes, regulation of transcription, DNA-dependent (GO:0006355;p=3.8×10^-12^), cell adhesion (GO:0007155;p=3.2×10^-5^) and chromatin modification (GO:0016568;p=0.0004) were the most enriched regulatory networks (Figure 2B, Supplementary Table 2B). In contrast, among the 717 genes co-regulated only by Slug, focal adhesion (p=8.05×10^-10^), ECM-receptor interaction (p=5.53×10^-8^) and pathways in cancer (p=1.06×10^-6^) were the top 3 enriched pathways (Figure 2B, Supplementary Table 2C). Biological processes such as cilium assembly (GO:0042384:p=1.06×10^-5^), microtubule-based movement (GO:0007018:p=1.07×10^-5^) and cell projection organization (GO:0030030:p=3.47×10^-5^) were observed for *Slug* co-regulated genes in GO analysis (Figure 2B, Supplementary Table 2D). This data suggests that Snail family members (Snail and Slug) regulate distinct cellular functions in ependymomas including cell proliferation, cellular motility and transcription regulation.

### *IL1R1*, a positive correlated gene regulated by both *Snail* and *Slug*, shows higher expression levels in ST-EPN-RELA and PF-EPN-A molecular subgroups

**Figure 3:**
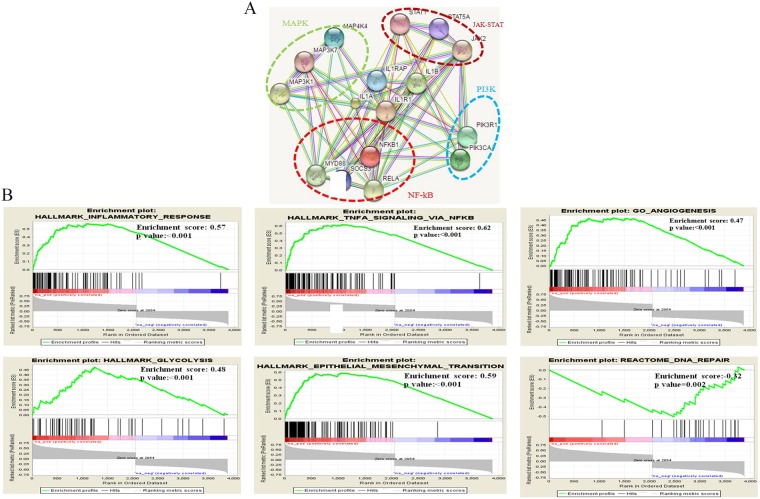
Regulation and pathway analysis of IL1R1 Protein-protein interaction (PPI) analysis of IL1R1 using STRING database **(A)**, GSEA analysis of IL1R1 co-expressed genes in RELA and PF-A subgroup from published dataset (Pajtler KW, et al. 2015) indicating inflammatory, EMT and angiogenesis related pathway to be enriched **(B)**.

The expression outcome of the 37 genes that were common to both Snail and Slug gene-sets were annotated by their expression status within Grade II and III ependymomas of GSE64415 dataset molecularly sub-grouped into ST-EPN-*RELA*, ST-EPN-*YAP1*, PF-EPN-A, PF-EPN-B and SP-EPN [6]. Among the 37 genes, the expression of IL1R1, a cytokine receptor belonging to the interleukin-1 receptor family, was selectively high in the most aggressive molecular subgroups viz. ST-EPN-*RELA* and PF-EPN-A leading us to explore *IL1R1* further. We first validated expression levels of *IL1R1* on in-house EPN samples and observed upregulated IL1R1 expression in ST-*RELA*+ subgroup as compared to ST-*REL*A- (p<0.001), and in PF-A group as compared to PF-B group (p<0.01) (Figure 2C). Next, we found a significant positive correlation between *IL1R1* expression and both Snail (R=0.437; p<0.001) and Slug (R=0.518; p<0.001) (Figure 2D), suggesting an association of *IL1R1* with EMT-like phenotype in EPNs. Prediction of protein-protein interactions (PPIs) of IL1R1 from an online database STRING (http://string.embl.de/) showed direct (physical) and indirect (functional) interactions of IL1R1 with components of NF-kB pathway (RELA, MYD88, NFKB1, SOCS3), JAK-STAT pathway (STAT1, STAT5, JAK2), P13K pathway (PIK3R1, PIK3CA) and MAPK pathway (MAP3K1, MAP3K7, MAP4K4) (Figure 3A), all of which have been previously implicated in the pathogenesis of intracranial EPNs [5–8, 30], pointing towards a central role for IL1R1 in various oncogenic and stemness related pathways in EPNs.

### Inflammatory, angiogeneic and EMT related factors are regulatory network for *IL1R1* in ST-EPN-RELA and PF-EPN-A ependymomas

The preferential high expression of *IL1R1* in ST-EPN-*RELA* and PF-EPN-A molecular subgroups led us to search for similarities in the transcriptional activation patterns of *IL1R1* by using GSEA within a dataset of ST-EPN-*RELA* and PF-EPN-A molecular subgroups [6]. Gene sets for inflammation (GO_ACTIVATION_OF_IMMUNE_RESPONSE, GO_CYTOKINE_MEDIATED_SIGNALING_PATHWAY, HALLMARK_INFLAMMATORY_RESPONSE, HALLMARK_TNFA_SIGNALING_VIA_NFKB), angiogenesis (GO_ANGIOGENESIS), epithelial-to-mesenchymal transition (HALLMARK_EPITHELIAL_MESENCHYMAL_ TRANSITION) and glycolysis (HALLMARK_GLYCOLYSIS) showed positive enrichment with IL1R1 expression, whileREACTOME_DNA_REPAIR showed negative enrichment with IL1R1 expression (Figure 3B).

### *IL1R1* is expressed in the ependymal lining and sub-ventricular zone of juvenile mice and correlates with neural stem cell markers

**Figure 4:**
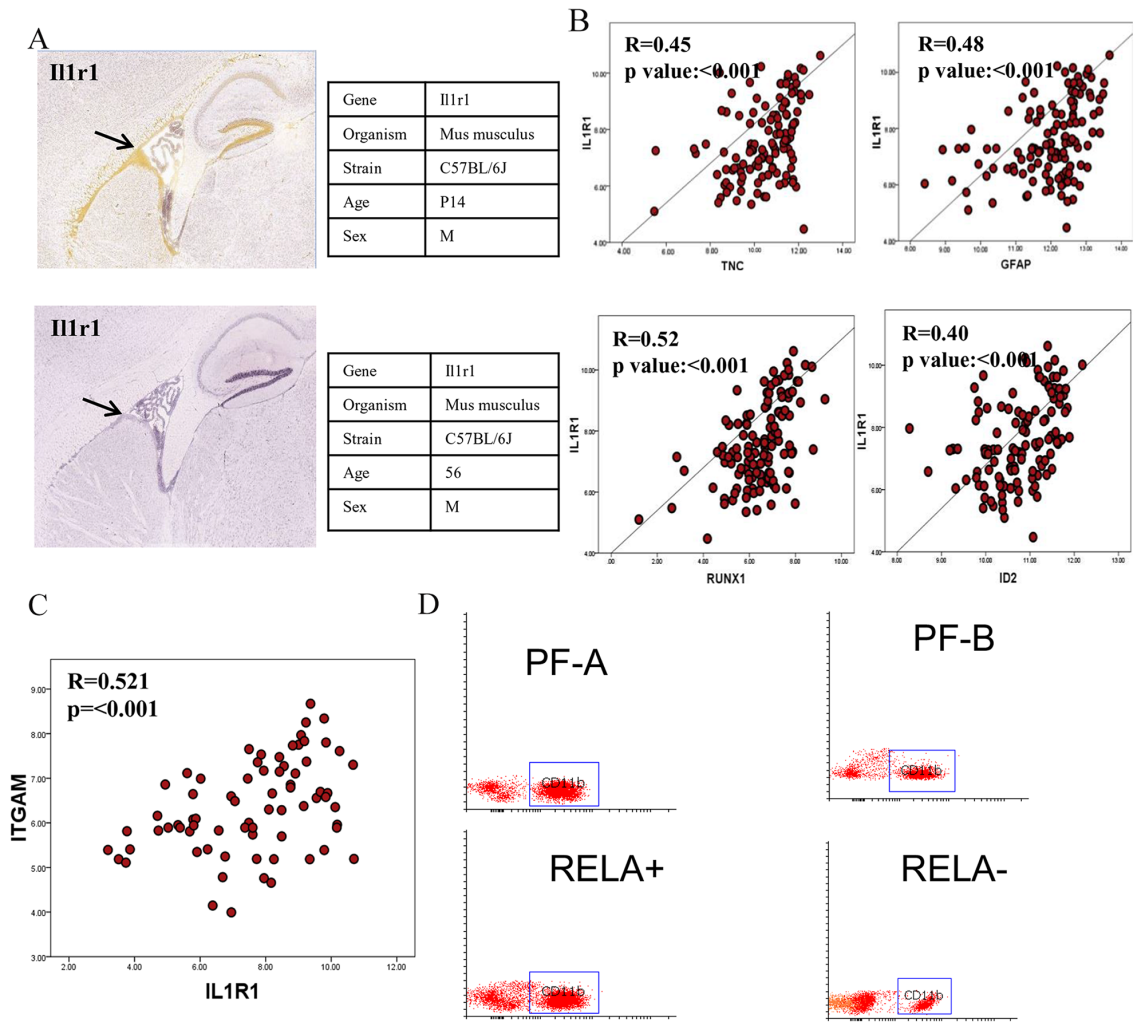
IL1R1 expression and its relation to neural stem cells and MDSC cells In-situ hybridization images for IL1R1 expression in developing and adult mice **(A)**, Correlation of IL1R1 expression with neural stem cells related genes **(B)**, Correlation plot between IL1R1 and ITGAM/ CD11b expression **(C)**, Flow cytometer analysis of CD11b cells in molecular subgroups of ependymomas **(D)**.

We analyzed publically available in-situ hybridization data from the Allen Developing Mouse Brain Atlas to investigate the anatomical distribution of *IL1R1* transcription. We found strong expression of *IL1R1* mRNA in ependymal lining and sub-ventricular zone in juvenile mice (P14), while there was loss of *IL1R1* mRNA in ependymal lining of adult mice (56 days) (Figure 4A). Considering that this subventricular zone is known to harbor stem cells capable of divergent differentiation [32] and radial-glia like stem cells are postulated to give rise to EPNs [5], we attempted to correlate *IL1R1* expression with known neural stem cell markers of subventricular zone such as GFAP, Tenascin C, ID2 and RUNX1 [5, 33–35]. Interestingly, a significant positive correlation was observed between *IL1R1* and *TNC* (R=0.45, p<0.001), *GFAP* (R=0.48, p<0.001), *RUNX1* (R=0.52, p<0.001) and *ID2* (R=0.40, p<0.001) on analyzing GSE64415 dataset [6] (Figure 4B). This suggests that *IL1R1* may play a role in differentiation and proliferation of neural stem cells.

### *IL1R1* correlates with increased numbers of tumor derived myeloid derived suppressor cells (CD11b^+^)

*IL1R1* is known to activate myeloid derived suppressor cells (MDSCs) through IL1β [36]. Gene expression data of ST-EPN-*RELA* and PF-EPN-A molecular subgroups of Pajtler et al [6] was analyzed for expression levels of *ITGAM*/CD11b^+^(marker of MDSCs cells) and correlated with *IL1R1* expression. A significant positive correlation was obtained between *IL1R1* and *ITGAM* (R=0.52, p<0.001) (Figure 4C). To validate this finding, FACS was performed for enumeration of CD11b^+^ cells in 16 in-house EPN tumor samples [ST-*RELA*+ (n=4), ST-*RELA*- (n=4), PF-A (n=4), PF-B (n=4)]. Interestingly, a significantly higher population of CD11b^+^ MDSCs was observed in ST-*RELA*+ cases as compared to ST-*RELA*- (p=0.021) and PF-A as compared to PF-B (p=0.003) (Figure 4D).

### *IL1R1* expression is higher in ependymomas as compared to other CNS tumors

**Figure 5:**
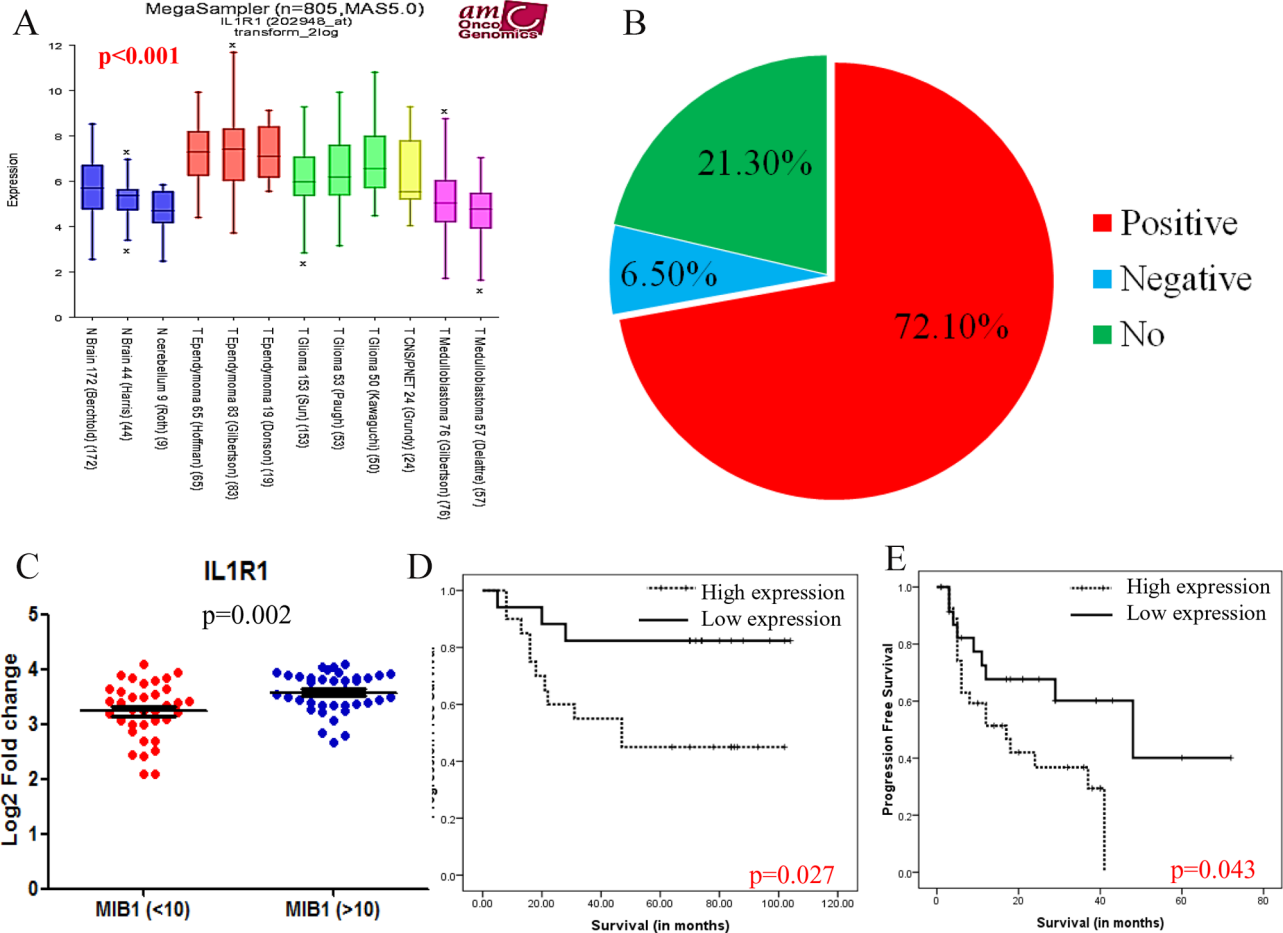
IL1R1 expression and its clinical importance Gene expression of IL1R1 expression in different CNS tumors and normal brain **(A)**, Pie chart for correlation of IL1R1 expression with EMT related genes **(B)**, Scatter plot of in-house IL1R1 expression with high (≥10%) and low (<10%) MIB1/Ki-67 labelling indices **(C)**, Univariate progression free survival analysis for IL1R1 expression in external cohort GSE27287 [7] **(D)** and in-house samples **(E)**.

We next analyzed *IL1R1* expression in other aggressive CNS tumors such as adult and pediatric gliomas, CNS/PNETs and medulloblastomas. Utilizing R2 software for selected GEO databases of different CNS tumors [37–41], as mentioned in methodology, we found that high expression levels of *IL1R1* were unique to EPNs (p<0.001) (Figure 5A). While slight elevations of *IL1R1* mRNA was noted in other gliomas as well, EPNs showed significantly higher expression levels (2 to 3 fold differences, p < 0.01). This selective expression of *IL1R1* in ependymomas suggests the possibility of *IL1R1* serving as a diagnostic marker for intracranial ependymomas and a potential therapeutic target.

### *IL1R1* significantly associates with EMT signatures

To gain deeper insights into the molecular functions of *IL1R1* in driving the aggressive phenotype in ependymomas, correlations were performed for *IL1R1* expression with published gene-sets of EMT signatures using gene expression data of Pajtler K, et al. [6] for Grade II and III EPNs. It was interesting to note that a high percentage of EMT genes (72.1%, 44/61), including *POSTN* (R=0.538, p=<0.001), *ADAM12* (R=0.633, p=<0.001) and *LOX* (R=0.337, p=<0.001), known to have an active role in activation and maintenance of EMT-like phenotype, positively correlated with *IL1R1* expression across 5 molecular subgroups (Figure 5B). Less than 10% of genes (6.5%, 4/61) showed significant negative correlation such as *THYN1* (R=-0.420, p=<0.001) and *SULF1* (R=-0.287, p=0.002), while the remaining 21.3% (13/61) genes showed no correlation with *IL1R1*.

### High *IL1R1* expression correlates with higher tumor cell proliferation rate and worse clinical outcome

IL1R1 expression significantly correlated with higher MIB1/Ki-67 labelling indices (≥10%) (p=0.002) (Figure 5C). On univariate analyses, we found significantly increased hazard ratios and poor disease-free survival in patients with high expression levels of *IL1R1* (HR=4.37, p=0.009) within the in-house EPN cohort (Figure 5D). Although multivariate analysis could not be performed due to small number of events in our cohort, we confirmed the poor prognosis of high IL1R1 expression in an independent external ependymoma cohort GSE27287 (p=0.027) [7] (Figure 5E).

## DISCUSSION

In the present study, we have utilized a co-expression based method to analyze messenger RNA expression data set for *Snail* and *Slug* genes, with an aim to identify genes correlating with clinical variables including patient outcome. To achieve this, we first performed in-silico gene expression analysis across 3 independent cohorts of EPNs [7, 30, 31] and found significant upregulation of Snail and Slug expression in intracranial EPNs. We then validated these findings by performing gene expression analysis on 75 in-house EPNs samples subdivided into five distinct clinico-pathologic-molecular subgroups and demonstrated maximal upregulation of Snail and Slug in pediatric PF-EPNs followed by *RELA* fusion-positive ST EPNs. Gene co-expression analysis for Snail and Slug performed on microarray dataset of Witt H et al [7] identified *IL1R1*, a cytokine receptor belonging to the interleukin-1 receptor family, to positively correlate with *Snail* and*Slug* expression, with highest enrichment in ST-EPN-*RELA* and PF-EPN-A molecular subgroups of Pajtler et al dataset [6]. Supportive of these findings, gene expression analysis of our in-house EPN cohort showed upregulated *IL1R1* expression levels in corresponding clinico-pathologic-molecular subgroups (ST-RELA+ and pediatric PF) with significant correlation with higher *Snail/Slug* expression levels, higher proliferative indices and poorer progression free survival. Further analysis into the functional significance of *IL1R1* in EPNs revealed selective upregulation in EPNs as compared to other common brain tumors, strong expression in subventricular zones of juvenile but not adult mice brain, and association with expression of neural stem cell markers. Significant interactions with inflammatory, angiogenic, glycolytic and EMT related pathways were noted within the ST-EPN-*RELA* and PF-A molecular subsets. In particular, we demonstrated higher numbers of MDSCs, a known target of IL1R1-IL1β signaling, in *RELA* fusion positive ST EPNs and pediatric PF EPNs of our cohort by FACS. Our study for the first time reports a role for the inflammatory marker, *IL1R1* of the interleukin-1 family, in ST-EPN-*RELA* and PF-EPN-A molecular subsets of EPNs, with possible roles in regulation of EMT, stemness, angiogenesis, metabolism and tumor inflammatory responses.

IL1R1/CD121a is an interleukin receptor which belongs to interleukin-1 cytokine receptor family. Physiologically, IL1R1 acts as a receptor for interleukin 1 alpha (IL1A), interleukin 1 beta (IL1B), and interleukin 1 receptor antagonist (IL1RA). Our PPI based analysis further identified its involvement in various oncogenic pathways including NF-kB, MAPK, PI3K which are known to be highly expressed in intracranial EPNs [6, 8, 42]. Notably, increased expression of IL1R1 regulates expression of several chemo- and radio resistant genes such as *COX2*, *ABCG2*, *PTGES* and *RAGE* [43]. Increased expression of COX2, leading to tumor growth and acquisition of multi-drug resistance has been previously reported in childhood EPNs [44, 45]. Overexpression of ABCG2 gene has also been reported in ependymomas [46, 47]. GSEA for *IL1R1* identified pathways related to inflammation and angiogenesis to be further enriched in ST-EPN-*RELA* and PF-EPN-A molecular subgroups. Supportive of our findings, previous studies have already implicated these pathways in the pathogenesis of EPNs [48–50]. Of note, DNA repair pathway was found to be negatively correlated with IL1R1 expression, indicating impairment of DNA repair in IL1R1 expressing EPNs. Considering that chromothripsis and epigenetic alterations are the hallmarks of ST-EPN-*RELA* [6] and PF-EPN-A [7] molecular subgroups respectively, the loss in the DNA repair mechanisms may be a contributing factor.

Ependymomas are postulated to originate from radial glia-like cancer stem cells [5]. Previous studies have demonstrated increased expression of stem cell marker nestin to be associated with poor prognosis in intracranial ependymomas [51], with high expression levels in *RELA* fusion positive ST EPNs [8]. Similarly, stem cell pathways such as NOTCH and high expression levels of the developmental extracellular matrix glycoprotein, Tenascin C, are implicated in the pathogenesis of pediatric posterior fossa EPNs and PF-EPN-A molecular subgroup [52]. In-situ hybridization for IL1R1 performed in our study localized its physiological expression in the subventricular zone of neonatal but not adult mice. Subventricular zone is known to harbor neural stem cells involved in active neurogenesis [32], strengthening our hypothesis that IL1R1 may be involved in stemness traits. In support of our finding, previous studies have found IL1R1 expression on Nestin/SOX2 positive neural progenitor cells [53]. Further, it is well known that EMT phenotype also confers stem cell traits [11, 12], and we demonstrated correlation of IL1R1 with upto 72% of EMT genes, suggesting common links among IL1R1, EMT phenotype and stemness.

A gene ontology based analysis of a small cohort of recurrent and non-recurrent pediatric EPNs identified that immune function related genes were enriched in non-recurrent EPNs and correlated with a better outcome [54]. In the same study, they demonstrated by immunohistochemistry that these genes are likely overexpressed by a subset of tumor infiltrating microglia/macrophages, which were significantly more populous in the non-recurrent EPNs as compared to their recurrent counterparts [54]. Although non-specific immunotherapeutic strategies have occasionally been tried in ependymomas [55], the lack of appropriate antigen targets has precluded development of valid immune-based therapies in EPNs [56]. In this context, IL1R1 emerges as a potential target. Tu S et al reported activation of myeloid derived suppressive cells (MDSCs) in tumor via IL1β, both *in-vitro* and *in-vivo*, through an IL1R/NF-kB pathway [36]. In the present study, quantification of CD11b+ cells using FACS demonstrated significantly higher levels of MDSCs in *RELA* fusion positive ST EPN and pediatric PF EPNs, further supported by the positive correlation of gene expression between *IL1R1* and *ITGAM*/ CD11b+ in ST-EPN-*RELA* and PF-EPN-A molecular subgroups of Pajtler KW et al [6]. This suggests that ILIR1 may play a role in immune escape. Further, MDSCs can also directly incorporate into tumor endothelium and promote tumor angiogenesis by producing high levels of matrix metalloproteinase-9 (MMP9) [36]. We found significant correlation of IL1R1 with MMP11 expression, which in involved in breakdown of extracellular matrix, tissue remodeling and metastasis. High microvascular densities and increased expression of vascular endothelial derived growth factor (VEGF) has been observed in intracranial EPNs [51] and our findings suggest that IL1R1 may play a role in these phenotypic changes as well.

A large number of studies have highlighted site specific variations in the cytogenetic, gene expression, molecular and epigenetic profiles of histologically similar appearing EPNs arising from different anatomical locations [3–7]. More recently, a large scale DNA methylation study identified nine molecular subgroups of EPNs within the three anatomical compartments viz. ST, PF and SP [6]. The two most common molecular subgroups: ST-EPN-*RELA* and PF-EPN-A comprise of Grade II/III tumors, predominate in children and associate with very poor outcome [6, 8]. While DNA methylation assay is considered gold standard for molecular subgrouping [6], surrogate immunohistochemical markers such as L1CAM and pRelA/p65 for ST-EPN-RELA, and LAMA2 and H3K27me3 for PF-EPN-A subgroup [6–8, 57–58] have been suggested. Limited studies have reported their utility and reproducibility in clinical practice and our co-expression study for *Snail* and *Slug* has identified a unique marker, *IL1R1,* to be highly expressed in these two subgroups and correlated with higher tumor cell proliferation rates. High IL1R1 expression also emerged as a poor prognostic marker for progression free survival in our in-house cohort and in an external independent cohort [7], strengthening the clinical importance and translational potential of our findings. Thus, IL1R1 presents with a potential to serve as a diagnostic marker and therapeutic target in these aggressive subgroups. Given the availability of selective inhibitors of IL1R1 [59], further investigations into the pharmacological inhibition of IL1R1 in ST-EPN-RELA and PF-EPN-A molecular subgroups are warranted.

To conclude, the present study identified IL1R1, an inflammatory cytokine, as a co-expressed gene for Snail and Slug in intracranial EPNs. ILIR1 was selectively upregulated in ependymomas, as compared to other common CNS tumors, with highest expression levels in the aggressive ST-EPN-*RELA* and PF-EPN-A molecular subgroups and showed positive correlation with EMT markers, higher proliferation index, and immunological and angiogenesis related pathways and stemness. IL1R1 expression associated with worse prognosis and further studies are warranted to evaluate and validate IL1R1 as a therapeutic target.

## MATERIALS AND METHODS

### Gene co-expression analysis for *Snail* and *Slug*

Gene co-expression analysis based on the microarray data generated by Witt H, et al (GSE27279) was performed [7]. The source data set was originally generated using Affymetrix Human Exon 1.0 ST Array and comprised of 102 samples of EPNs of all sites (ST-31; PF-54, SP-15; site not available in 2 samples). The data was analyzed using database analysis tool R2: Genomics Analysis and Visualization Platform (http://r2.amc.nl) and normalized using the MAS 5.0 algorithm. To start with, we employed Pearson's correlation coefficient and computed genes associated with Snail (*SNAI1*) and Slug (*SNAI2*) expression in all EPN cases. Next, in order to select significant gene subsets showing strong correlation (positive and negative) with Snail and Slug expressions and to shrink the gene list to a manageable size, we setup a filter to select only for genes showing an absolute correlation cutoff greater than ±0.3 Pearson's value and p value less than 0.05. Repetitive genes were removed while retaining only one representative gene with most significant p-value among replicates. We next assessed Gene Ontology (GO) terms and KEGG pathways using hypergeometric tests for these transcripts. P-values were adjusted for multiple testing using Benjamini–Hochberg correction. Patient outcome data was also retrieved for this EPN cohort (GSE27279) for survival analysis [7].

### In-house ependymoma sample collection

The study was of ambispective design (2003-2016) and ethically approved by the Institute Ethics Committee (Ref No: IESC/T-211/05/05/2015). Tumor samples with a diagnosis of ependymoma, as reconfirmed independently by two neuropathologists (MCS & AN) and classified according to the 2016 World Health Organisation (WHO) classification of CNS tumors [1], with available tumor tissue, including snap-frozen tissue and clinical follow-up, were included for analysis. WHO Grade I EPNs (subependymomas and myxopapillary ependymomas) were excluded. Clinical follow-up data was obtained from the records of the department of Neurosurgery.

#### Clinico-pathologic-molecular subgrouping of cases

Quantitative real time polymerase chain reaction (qRT-PCR) was performed for the detection of Type 1 and 2 *C11orf95-RELA, YAP1-MAMLD1* and *YAP1-FAM118B* fusion transcripts in ST EPNs using cDNA as described previously [8]. ST EPNs with *RELA* fusions were grouped as ST-RELA+ while those with *YAP1* fusions were grouped as ST-*YAP1*+ and the remaining grouped as ST-RELA-. Among PF EPNs, those occurring in children ≤18 years of age were grouped as PF-A, while those in adults >18 years were grouped as PF-B. Spinal Grade II/III ependymomas were grouped as SP group.

#### Gene expression analysis for Snail, Slug and IL1R1 on in-house ependymoma tumor samples

Total RNA was isolated using mirVana™ miRNA Isolation Kit (M/S Ambion, Life Technologies, USA) as per manufacturer's protocol. One μg of total RNA was reverse transcribed using Superscript VILO cDNA Synthesis Kit (M/S Invitrogen, Life Technologies, USA). Quantitative Real time PCR (qPCR) was performed using Syber-green with Agilent Mx3005P system (Agilent Technologies, USA). The differences in expression were calculated using the comparative method and the level of Snail, Slug and IL1R1 fold change was calculated using 2−ΔΔCt method using *ACTB* and *GAPDH* as housekeeping genes. The primer sequences for the transcripts analyzed are provided in Supplementary Table 3.

### Microarray datasets of independent published ependymoma cohorts for in-silico data analysis

Gene expression microarray dataset of three independent cohorts of EPNs: GSE21687 (n=83) [30], GSE27279 (n=102) [7] and GSE50385 (n=65) [31] were analyzed for the expression levels of Snail and Slug using R2 software. For comparison of *IL1R1* expression between various CNS tumors, 2 gene expression microarray datasets for medulloblastoma: GSE37418 (n=76) [37] and GSE NA, 1 dataset for CNS-PNETs: GSE19404 (n=23) [38], 3 dataset for gliomas: GSE43378 (n=50) [39], GSE19578 (n=53) [40] and GSE4290 (n=157) [41], and 3 normal brains: GSE7307 (n=1), GSE13564 (n=1) [60] and GSE11882 (n=1) [61], were analyzed using R2 software.

Detailed methodology for FACS for CD11b quantification, GSEA analysis, immunohistochemistry for MIB1 and ISH data can be found in Supplementary materials and methods.

### Statistical analysis

Kaplan–Meier survival analysis was used to obtain survival estimates. GraphPad Prism version 5.0 for Windows was used for constructing scatter, correlation and box plots. Pearson correlation, ANOVA and *T*-test were performed using SPSS version 11.5 for Windows. In all statistical analyses, 2-sided test with P value less than 0.05 was considered as significant.

## SUPPLEMENTARY MATERIALS TABLES




