# Supplementary material for: Transcriptional co-expression regulatory network analysis for Snail and Slug identifies IL1R1, an inflammatory cytokine receptor, to be preferentially expressed in ST-EPN-RELA and PF-EPN-A molecular subgroups of intracranial ependymomas
Source: Oncotarget. 2018 Oct 26;9(84):35480–92. doi: 10.18632/oncotarget.26211 (PMC6231457; doi:10.18632/oncotarget.26211)
Supplement: Supplementary file 1 [file oncotarget-09-35480-s001.pdf]

Transcriptional co-expression regulatory network analysis for Snail and Slug identifies *IL1R1,* an inflammatory cytokine receptor, to be preferentially expressed in ST-EPN-*RELA* and PF-EPN-A molecular subgroups of intracranial ependymomas

SUPPLEMENTARY MATERIALS

Quantification of CD11b expressing tumor cells using Fluorescence assisted cell sorting (FACS)

Fresh/Frozen tumor tissue samples were collected in F-10 media (Thermo fisher, USA) and immediately disaggregated using surgical blade. A single-cell suspension was obtained using collagenase and was washed with phosphate buffered saline twice. FACS was performed on BD FACSAria III flow cytometer (BD Biosciences, USA) and analysis was performed by BD FACSDiva version 6.1.3 software. Samples were primarily gated on the basis of size (forward-scatter) and granularity (side-scatter), followed by exclusion of dead cells.

Gene set enrichment analysis (GSEA)

GSEA was performed as described previously [32], to compute *IL1R1* significant pathways in ST-EPN-*RELA* and PF-EPN-A molecular subgroups utilizing microarray data of GSE64415 [6].

Correlation of EMT gene signatures with *IL1R1* expression

EMT signatures derived from the published dataset of Anastassiou D et al (33) were analyzed using GSE64415 data-set [6].

In-situ hybridization data of IL1R1

Allen Brain Atlas (http://developingmouse.brain-map.org/), an anatomical atlas was utilized as a resource that provides spatial representations of brain. Images for IL1R1 expressions were obtained for P14 and 56 days mice.

Immunohistochemistry for MIB1

Immunohistochemistry was performed on 5-µm thick formalin-fixed, paraffin-embedded tumor sections using antibodies directed against MIB-1/Ki-67 (Dako, Denmark; 1:200). Labeled streptavidin biotin kit (Universal) was used as a detection system (Dako, Denmark). MIB-1 labelling index was calculated as percentage after counting 1000 tumor cell nuclei in different hot spots.

Supplementary Table 1: Clinico-pathological features of the selected cases of ependymomas

Variable		Number of cases (%)
**Total number of cases**		75(100%)
**Gender**		
Male		51 (68%)
Female		24 (32%)
**Age**		
Pediatric (<18 years)		51 (68%)
Female (>18 years)		24 (32%)
	**Molecular Groups of 75 ependymoma cases**	
**Supratentorial (n=36)**	ST- RELA+	23 (63.8%)
	ST- RELA-	13 (36.1%)
**Infratentorial (n=31)**	PF-A	19 (61.2%)
	PF-B	12 (38.7%)
**Spinal (n=8)**	SP	8 (100%)

Supplementary Table 2A: List of KEGG pathways co-regulated by Snail with p-value less than 0.05 (dataset used Mack SC, et al. 2011)

Serial no	Annotations	Hypergeometric p value	Genes
1	(KEGG) 04380: Osteoclast differentiation	0.0001	12 genes
2	(KEGG) 04350: TGF-beta signaling pathway	0.019	7 genes
3	(KEGG) 00562: Inositol phosphate metabolism	0.024	6 genes
4	(KEGG) 04070: Phosphatidylinositol signaling system	0.045	6 genes

Supplementary Table 2B: List of Biological processes co-regulated by Snail with p-value less than 0.05 (dataset used Mack SC, et al. 2011)

Serial no	Annotations	Hyper-geometric p value	Genes
1	GO:0006355: regulation of transcription, DNA-dependent (BP)	3.08E-12	73 genes
2	GO:0007155: cell adhesion (BP)	3.20E-05	29 genes
3	GO:0016568: chromatin modification (BP)	0.000424052	16 genes
4	GO:0046777: protein autophosphorylation (BP)	0.0370075	10 genes
5	GO:0006298: mismatch repair (BP)	0.0383494	4 genes
6	GO:0007165: signal transduction (BP)	0.0458928	36 genes
7	GO:0042493: response to drug (BP)	0.0471049	15 genes

Supplementary Table 2C: List of KEGG pathways co-regulated by Slug with p-value less than 0.05 (dataset used Mack SC, et al. 2011)

Serial no	Annotations	Hyper-geometric p value	Genes
1	(KEGG) 04510: Focal adhesion	8.00E-10	23 genes
2	(KEGG) 04512: ECM-receptor interaction	5.50E-08	14 genes
3	(KEGG) 05200: Pathways in cancer	1.10E-06	24 genes
4	(KEGG) 04666: Fc gamma R-mediated phagocytosis	0.00044	10 genes
5	(KEGG) 04670: Leukocyte transendothelial migration	0.00212	10 genes
6	(KEGG) 05146: Amoebiasis	0.0042	9 genes
7	(KEGG) 04810: Regulation of actin cytoskeleton	0.00429	13 genes
8	(KEGG) 04640: Hematopoietic cell lineage	0.00455	8 genes
9	(KEGG) 05100: Bacterial invasion of epithelial cells	0.00711	7 genes
10	(KEGG) 04974: Protein digestion and absorption	0.01126	7 genes
11	(KEGG) 04360: Axon guidance	0.01133	9 genes
12	(KEGG) 04380: Osteoclast differentiation	0.01215	9 genes
13	(KEGG) 05410: Hypertrophic cardiomyopathy (HCM)	0.01285	7 genes
14	(KEGG) 05140: Leishmaniasis	0.0163	6 genes
15	(KEGG) 05414: Dilated cardiomyopathy	0.0169	7 genes
16	(KEGG) 05150: Staphylococcus aureus infection	0.01705	5 genes
17	(KEGG) 04610: Complement and coagulation cascades	0.01707	6 genes
18	(KEGG) 04270: Vascular smooth muscle contraction	0.018	8 genes
19	(KEGG) 05412: Arrhythmogenic right ventricular cardiomyopathy (ARVC)	0.02323	6 genes
20	(KEGG) 04722: Neurotrophin signaling pathway	0.02384	8 genes
21	(KEGG) 04662: B cell receptor signaling pathway	0.02536	6 genes
22	(KEGG) 04146: Peroxisome	0.02587	6 genes
23	(KEGG) 04530: Tight junction	0.02634	8 genes
24	(KEGG) 04144: Endocytosis	0.02684	10 genes
25	(KEGG) 04060: Cytokine-cytokine receptor interaction	0.0272	12 genes
26	(KEGG) 00564: Glycerophospholipid metabolism	0.0276	6 genes
27	(KEGG) 04614: Renin-angiotensin system	0.02783	3 genes
28	(KEGG) 05222: Small cell lung cancer	0.03331	6 genes
29	(KEGG) 04540: Gap junction	0.03766	6 genes
30	(KEGG) 04020: Calcium signaling pathway	0.03767	9 genes
31	(KEGG) 04012: ErbB signaling pathway	0.03811	6 genes

Supplementary Table 2D: List of Biological processes co-regulated by Snail with p-value less than 0.05 (dataset used Mack SC, et al. 2011).

See Supplementary File 1

Supplementary Table 3 : List of primers used for Gene expression studies

Gene of interest	FORWARD PRIMER	REVERSE PRIMER
**SNAIL**	5'-GAGGCGGTGGCAGACTAG-3'	5-GACACATCGGTCAGACCAG-3'
**SLUG**	5'-CATGCCTGTCATACCACAAC-3'	5'-GGTGTCAGATGGAGGAGGG-3'
**IL1R1**	5'-ATTGATGTTCGTCCCTGTCC-3'	5'-TGAATCCTGGAGGCTTGTTC-3'
**ACTB**	5'-GCCGGGACCTGACTGACTAC-3'	5'-TTCTCCTTAATGTCACGCACGAT-3'
**GAPDH**	5'-ACCCACTCCTCCACCTTTGAC-3'	5'-TGTTGCTGTAGCCAAATTCGTT-3'
